# Prevalence of systemic venous congestion assessed by Venous Excess Ultrasound Grading System (VExUS) and association with acute kidney injury in a general ICU cohort: a prospective multicentric study

**DOI:** 10.1186/s13054-023-04524-4

**Published:** 2023-06-08

**Authors:** Stefan Andrei, Pierre-Alain Bahr, Maxime Nguyen, Belaid Bouhemad, Pierre-Grégoire Guinot

**Affiliations:** 1 Department of Anaesthesiology and Critical Care Medicine, Dijon University Medical Centre, 21000 Dijon, France; 2 Department of Anaesthesiology and Critical Care Medicine, University of Medicine and Pharmacy “Carol Davila”, Bucharest, Romania; 3 LNC UMR1231, University of Burgundy and Franche-Comté, 21000 Dijon, France

**Keywords:** ICU, Sepsis, Cardiac, Ultrasound, Congestion, VExUS, POCUS, Acute kidney injury, Mortality

## Abstract

**Background:**

The importance of assessing venous congestion in ICU patients is widely acknowledged, but its study is hampered by the lack of a practical evaluation tool. The Venous Excess Ultrasound Grading System (VExUS), based on a semi-quantitative combined ultrasound assessment, has been associated with acute kidney injury (AKI) in cardiac ICU patients. The objectives of this study were to assess the prevalence of congestion using VExUS in general ICU patients, and to evaluate the association between VExUS, AKI and death.

**Methods:**

This prospective, observational study included adult patients within 24 h of ICU admission. VExUS and hemodynamic parameters were measured four times during the ICU stay: within 24 h of ICU admission, after day 1 (between 24 and 48 h), after day 2 (between 48 and 72 h), and last day of ICU stay. The prevalence of AKI during the first week in ICU and 28-day mortality were assessed.

**Results:**

Among the 145 patients included, the percentage of patients with a VExUS score of 2 (moderate congestion) and 3 (severe congestion) was 16% and 6%, respectively. The prevalence did not change over the study period. There was no significant association between admission VExUS scores and AKI (*p* = 0.136) or 28-day mortality (*p* = 0.594). Admission VExUS ≥ 2 was not associated with AKI (OR 0.499, CI_95%_ 0.21–1.17, *p* = 0.109) nor 28-day mortality (OR 0.75, CI_95%_ 0.2–2.8, *p* = 0.669). The results were similar for VExUS scores measured at day 1 and day 2.

**Conclusions:**

In general ICU cohort the prevalence of moderate to severe venous congestion was low. Early assessment of systemic venous congestion using VExUS scores was not associated with the development of AKI or with 28-day mortality.

**Supplementary Information:**

The online version contains supplementary material available at 10.1186/s13054-023-04524-4.

## Clinical perspective

What is new?

- Prevalence and clinical significance of systemic venous congestion assess by the venous excess ultrasound score (VExUS) is unknow in general ICU
- Our study revealed a low prevalence of venous congestion and did not demonstrate any association between VExUS and acute kidney injury in general ICU patients.
- The utility of VExUS in all ICU patients should be reconsidered, but further studies are needed to evaluate the interest of VExUS in specific ICU populations.

## Introduction

Fluid overload is associated with higher morbidity and mortality in ICU patients [1, 2]. One underlying pathophysiological mechanism may be the venous congestion that is caused by fluid overload and cardiac dysfunction (right/left, systolic/diastolic), further impairing organ and tissue perfusion [3, 4]. Venous congestion has been studied extensively in cardiology in the context of cardio-renal syndrome, cardio-hepatic syndrome, and intestinal dysfunction [3, 5, 6]. While the importance of prevention, diagnosis and treatment of congestion is now widely acknowledged, the real prevalence of systemic venous congestion has never been assessed in general ICU patients. There are insufficient data regarding the prevalence of venous congestion and its association with outcomes in ICU patients.

One notable reason for this lack of data is the dual challenge of establishing a well-standardized definition for venous congestion and of developing a tool to evaluate it. Several venous ultrasound-based parameters (i.e. the inferior vena cava diameter, the portal pulsatility index, the renal venous flow pattern) have been proposed [7–9]. These parameters have been associated with outcomes such as acute kidney injury (AKI) [10–12]. Based on these venous ultrasound parameters, some authors have proposed a semi-quantitative grading score of venous congestion: the Venous Excess Ultrasound Grading System (VExUS) [13]. The VExUS is a scoring system that quantifies systemic venous congestion by using ultrasound examination of the inferior vena cava diameter, the sus-hepatic vein flow, the portal pulsatility index, and the renal venous flow pattern [13]. The VExUS was initially developed in the specific context of cardiac surgery [12], where it was demonstrated to be associated with the occurrence of AKI. Until now, only one paediatric study has evaluated the association between VExUS and elevated central venous pressure [14]. No study using the VExUS has been performed in adult general ICU. Thus, there is no data on VExUS and its association with outcomes in ICU population.

The primary aim of this study was to describe the prevalence of venous congestion based on the VExUS in general ICU patients. The secondary outcomes were to evaluate the association between VExUS, AKI, and 28-day mortality.

## Methods

### Patients and study design

The study protocol received the approval of an independent ethics committee (IRB 2020-A01819-30, CPP Sud Ouest Outre Mer III, ClinicalTrials.gov Identifier: NCT04680728). We conducted this prospective, observational study in four ICUs of university-affiliated and tertiary hospitals from October 2020 to October 2022. The patient or their kin provided written informed consent for study participation. The study was conducted in accordance with the 1964 Declaration of Helsinki. We included adult patients within 24 h of ICU admission. The exclusion criteria were: (1) person not affiliated to national health insurance, (2) age < 18 years, (3) pregnant or breastfeeding women, (4) poor echogenicity confirmed by the ultrasound operator, (5) chronic atrial fibrillation, (6) mechanical cardiac assistance, and (7) uncontrolled blood pressure (MAP < 65 mmHg).

### Data collection

Patients underwent clinical, biological, echocardiographic, and venous ultrasound assessments at several points in time: ICU admission (first 24 h), day 1 (24–48 h), day 2 (48–72 h), and the last day of ICU stay. The patients’ demographic characteristics (age, gender, weight, BMI), comorbidities, the reason for admission, and SAPS II scores were collected at inclusion. The clinical assessment included haemodynamic parameters [blood pressure, heart rate, central venous pressure (CVP)], diuresis, 24-h hydric balance, use of renal replacement therapy, ventilatory parameters (invasive or non-invasive mechanical ventilation use, FiO_2_, tidal volume, respiratory rate, end expiratory pressure), type of shock (sepsis, postoperative, haemorrhagic, cardiogenic), catecholamines (type, dose, duration), and mortality. The biological (laboratory) parameters included: haemoglobin, haematocrit, sodium, arterial lactate, and creatinine.

### Ultrasound measurements

The VExUS score was calculated as previously described [13]. We quantified the type C of the VExUS because it demonstrated an association with AKI for grade ≥ 2 [12, 13]. The ultrasound measurements were performed according to the current guidelines [8, 15, 16]. The acquisition of ultrasound images was performed by a board-certified physician using the Philips Envisor ultrasound system (Affinity ultrasound system Philips Medical System, Suresnes, France) and concomitant ECG. The ultrasound parameters were calculated as the average of five measurements (if not specified otherwise), regardless of the respiratory cycle. Data were acquired, stored, and reviewed offline by an experienced operator blinded to the study outcomes.

The diameter of the inferior vena cava (IVC) was measured in the subcostal view at 1.0 cm from its junction with the right atrium, with the patient in the supine position. The maximum and minimum diameters of the IVC were measured, and the percentage of the change in diameter was calculated. The portal vein flow pulsatility index (PI) was assessed by performing a pulsed-wave (PW) Doppler in the liver hilum [17]. The maximum velocity (Vmax) and minimum velocity (Vmin) of the portal vein were also measured, allowing the calculation of portal PI with the following formula: PI = (Vmax − Vmin)/(Vmax). PW Doppler measurements were performed in the interlobar veins of the upper, median and lower segments of each kidney [12]. The intrarenal venous flow was classified according to the waveform pattern [12, 18].

### Definitions and outcomes

The study primary endpoint was the VExUS score. It was calculated four times during the ICU stay: within 24 h of ICU admission, after day 1 (between 24 and 48 h), after day 2 (between 48 and 72 h), and at ICU discharge. The patients were classified as being congestive (VExUS ≥ 2) or not being congestive (VExUS < 2) [13]. A VExUS grade 2 was defined as moderate congestion, and a VExUS grade 3 was defined as severe congestion [13].

The secondary endpoints were AKI during the first week in ICU and 28-day mortality. AKI was defined according to Kidney Disease Improving Global Outcomes (KDIGO) criteria. AKI stage 1 is defined as increase in serum creatinine (SCr) by ≥ 0.3 mg/dL or increase in SCr to ≥ 1.5 times baseline, or urine output < 0.5 mL/kg/h for 6–12 h. AKI stage 2 is defined as increase in SCr to ≥ 2.0–2.9 times the baseline, or urine output < 0.5 mL/kg/h for ≥ 12 h. AKI stage 3 is defined as a SCR of up to 3.0 times the baseline or SCR increased to ≥ 4.0 mg/dL or initiation of renal replacement therapy or urine output < 0.3 mL/kg/h for ≥ 24 h or anuria for ≥ 12 h [19]. Baseline SCr was determined using the value obtained within a 3-month period before ICU admission (from hospital medical records, family physician records) and considered to be representative of the baseline kidney function according to the treating physician [20]. If no baseline creatinine value was available and patients had no documented history of renal disease, the baseline creatinine level was estimated to be 1 mg/dL. The patients with AKI at ICU admission were considered to have AKI at the moment of ICU admission. All outcomes were collected at the time of hospital discharge, death was noted within 28 days of admission to the ICU.

### Statistical analyses

Based on previous studies assuming the prevalence of venous congestion being around 15%, and the prevalence of AKI being of 45%, it was estimated that a cohort of 145 patients would be sufficient to identify an association between VExUS and AKI with a statistical power of 80% and a significance level of 95% [10, 11, 14]. The data are presented as means (standard deviation), medians (25%;75% interquartile range), or numbers (percentage), as appropriate. The quantitative variables are compared using the Student’s t-test or the Mann–Whitney test, as appropriate. The proportions were compared using the chi-square or Fisher test, as appropriate. The association between the VExUS and outcomes (AKI, 28-day mortality) was evaluated using logistic regression models. All analysed patients for the primary objective had complete ultrasound measurements. For later timepoint analyses, we excluded the patients with incomplete echographic assessments, due to poor echogenicity, ICU discharge or death. The VExUS measured within 24 h of ICU admission (admission VExUS) was used as a categorial variable (0, 1, 2, 3). The higher VExUS scores were compared with VExUS 0 scores. Sensitivity analyses were performed by dichotomizing the VExUS (VExUS ≥ 2 versus VExUS < 2), and using the scores measured at admission, on day 1 and on day 2 after ICU admission. The dichotomization was based on Beaubien-Souligny et al. [13] study that demonstrated an association between AKI and VExUS (type C) grade ≥ 2. Furthermore, several Cox regression sensitivity analyses were performed. Statistical associations were evaluated at time of the events, thus patients who developed events (AKI or death) before the day of evaluation were excluded from these analyses. The sensitivity analyses were completed by a mixed-effects logistic model with AKI as the dependent variable, the patients as random effect, and the time and VExUS as fixed effects. The statistical analyses were performed using R software version 3.4.4 (R Foundation for Statistical Computing, Austria). A bilateral *p *value < 0.05 was considered statistically significant.

## Results

One-hundred eighty-five patients were included in the study; 40 (20%) were excluded due to incomplete echocardiographic data at inclusion (Additional file 1: Figure S1). The mean age was 64 (± 15) years with a median SAPS II score of 46 [37;59], and a median ICU LOS of 6 [4;13] days. The most frequent reason for admission to ICU was medical (92 patients, 63%): sepsis (31 patients, 21%), respiratory conditions (29 patients, 20%), and neurological conditions (21 patients, 15%). Patients’ demographic characteristics are presented in Table 1. The mean admission serum lactate was 2.4 (± 3) mmol/L.

**Table 1:** Study cohort’s general characteristics

Variables	Overall cohort (n = 145)
Age (years), mean (SD)	64 (15)
Gender, n (%)	
Males	86 (59%)
Body mass index (kg/m^2^), mean (SD)	27.6 (6)
Comorbidities, n (%)	
Chronic obstructive pulmonary disease	14 (10%)
High blood pressure	86 (59%)
Ischemic heart disease	49 (34%)
Chronic systolic heart failure	21 (15%)
Valvular heart disease	37 (26%)
Peripheral artery disease	12 (8%)
Diabetes on oral antidiabetics	26 (18%)
Diabetes on insulin	15 (10%)
Chronic kidney disease	15 (10%)
Stroke	15 (10%)
Medical ICU admission, n (%)	92 (63%)
Admission SAPS II, median [IQR]	46 [37;59]
Patients treated with catecholamines, n (%)	
Norepinephrine (yes)	63 (43%)
Dobutamine (yes)	11 (7%)

*ICU* intensive care unit, *n* number, *SAPS II* simplified acute physiology score II, *SD* standard deviation

### General description of the VExUS

**Fig. 1:**
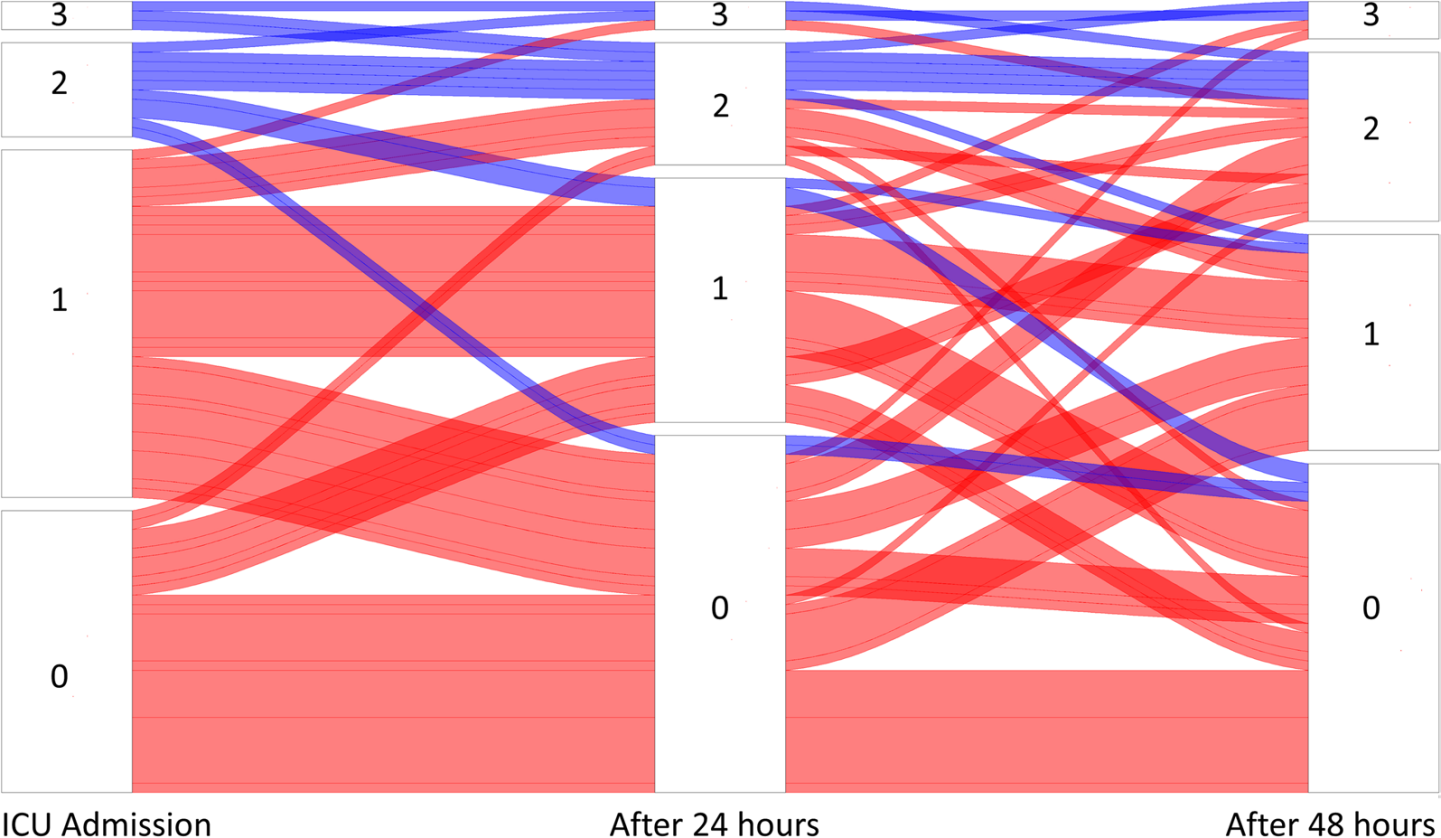
Alluvial diagram showing the profiles of evolution of VExUS during the first 72 h in ICU. Blue color—patients with admission VExUS ≥ 2. Red color—patients with admission VExUS < 2

Patients’ VExUS scores during the first 72 h since ICU admission and the fluctuations in scores over time are presented in Fig. 1. The VExUS categories at each timepoint during the first 72 h since ICU admission are presented in Table 2. The percentage of patients with a VExUS score of 3 was stable at around 6%. The percentage of patients with VExUS scores of 2 was 16% (23 patients), and VExUS scores of 3 was 4% (6 patients) at ICU admission, and the scores remained stable during the first 72 h in the ICU (Table 2, Fig. 1). Patients’ VExUS scores since ICU admission until ICU discharge and the fluctuations in scores over time are presented in Additional file 1: Figure S2. Patients’ baseline characteristics according to the VExUS scores are presented comparatively in Additional file 1: Table S1.

**Table 2:** VExUS scores in the ICU at different timepoints

Variables	Admission (n = 145)	Day 1 (n = 136)	Day 2 (n = 111)	Discharge (n = 96)
VExUS				
No congestion: 0	62 (43%)	68 (51%)	60 (54%)	68 (71%)
Mild congestion: 1	54 (37%)	40 (30%)	23 (21%)	18 (19%)
Moderate congestion: 2	23 (16%)	18 (13%)	22 (20%)	7 (7%)
Severe congestion: 3	6 (4%)	8 (6%)	6 (6%)	3 (3%)
Portal pulsatility index, n (%)				
Mild pulsatile	66 (45%)	53 (40%)	40 (36%)	36 (38%)
Severe pulsatile	31 (21%)	17 (13%)	18 (16%)	10 (11%)
Hepatic vein Doppler waveforms, n (%)				
Mild	50 (34%)	38 (28%)	31 (28%)	43 (45%)
Severe	18 (12%)	17 (13%)	13 (12%)	4 (4%)
Renal vein Doppler pattern, n (%)				
Mild	81 (56%)	42 (31%)	30 (27%)	9 (10%)
Severe	0 (0%)	24 (18%)	18 (16%)	9 (10%)
IVC diameter > 2 cm, n (%)	83 (57%)	66 (49%)	51 (46%)	28 (30%)
24 h hydric balance (mL), median [IQR]	990 [194;1880]	180 [− 1032;1161]	− 160 [− 1044;1693]	105 [− 620;2452]
Heart rate (bpm), median [IQR]	80 [67;97]	79 [69;95]	79 [70;93]	82 [72;99]
MAP (mmHg), median [IQR]	84 [72;93]	82 [73;92]	84 [75;93]	89 [79;96]
Cardiac output (L/min), median [IQR]	4.6 [3.6;5.7]	4.9 [3.9;5.8]	4.8 [4;5.8]	5.2 [4.2;6.2]
CVP (mmHg), median [IQR]	13 [9;15]	10 [8;14]	10 [7;12]	8 [8;12]
SV change following PLR > 10%, n (%)	31 (21%)	32 (24%)	21 (19%)	17 (18%)

*bpm* beats per minute, *CVP* central venous pressure, *ICU* intensive care unit, *n* number, *IVC* inferior vena cava, *MAP* mean arterial pressure, *PLR* passive leg raise test, *SAPS II* simplified acute physiology score II, *SD* standard deviation, *SV* stroke volume, *VExUS* venous excess ultrasound score

### Association between the VExUS and AKI

Of the 145 patients included, 68 (47%) patients developed AKI; 19 patients (13%) were classified as KDIGO III, 17 patients (12%) KDIGO II, and 32 patients (22%) KDIGO I. Twelve patients (8%) required renal replacement therapy.

The logistic regression did not demonstrate any association between admission VExUS and AKI (*p* = 0.136) (Table 3). An admission VExUS score ≥ 2 was not statistically associated with AKI (OR 0.499, CI_95%_ 0.21–1.17, *p* = 0.109). The VExUS score at day 1 was not associated with AKI (*p* = 0.372) (Table 3). VExUS ≥ 2 at day 1 was not associated with AKI (OR 0.35, CI_95%_ 0.11–1.15, *p* = 0.084). The results were similar for the VExUS at day 2 (*p* = 0.544) (Table 3). The VExUS ≥ 2 at day 2 was not associated with AKI (OR 0.41, CI_95%_ 0.1–1.7, *p* = 0.215). The lack of association between the risk of AKI and VExUS was confirmed by sensitivity analyses (Additional file 1: Table S2A and S2B and Additional file 1: Table S3A and S3B).

**Table 3:** Logistic regression results with AKI as dependent variable and VExUS at different timepoints as covariate

Variables	Odd ratio	95% confidence interval	*p* value
Admission VExUS (n = 145)			0.136
0	Reference	Reference	Reference
1	0.5	0.24–1.1	0.087
2	0.33	0.1–0.9	0.035
3	0.75	0.14–4.1	0.73
VExUS at day 1 (n = 108)^a^			0.372
0	Reference	Reference	Reference
1	0.98	0.4–2.38	0.960
2	0.37	0.1–1.5	0.170
3	0.27	0.1–2.5	0.254
VExUS at day 2 (n = 67)^a^			0.544
0	Reference	Reference	Reference
1	0.60	0.13–2.8	0.517
2	0.29	0.1–1.6	0.150
3	0.67	0.1–7.5	0.742

*VExUS* venous ultrasound excess score

^a^Patients who have developed AKI before the day of ultrasound examination were excluded

### Association between VExUS and 28-day mortality

Of the 145 patients, 39 (28%) patients were deceased at 28 days. Higher admission VExUS scores were not associated with 28-day mortality (Table 4) (*p* = 0.594). A VExUS ≥ 2 at ICU admission was not associated with 28-day mortality (OR 0.75, CI_95%_ 0.2–2.8, *p* = 0.669). No association was observed between VExUS scores at day 1 and 28-day mortality (*p* = 0.955) (Table 4). A VExUS ≥ 2 at day 1 was not associated with 28-day mortality (OR 0.47, CI_95%_ 0.1–2.2, *p* = 0.339). The results were similar for the VExUS at day 2 (Table 4), *p* = 0.955). The lack of association between the risk of 28-day mortality and VExUS were confirmed by sensitivity analyses (Additional file 1: Table S4A and S4B).

**Table 4:** Logistic regression results with 28-day mortality as dependent variable and VExUS at different timepoints as covariate

Variables	Odd ratio	95% confidence interval	*p*-value
Admission VExUS (n = 145)			0.594
0	Reference	Reference	Reference
1	0.5	0.1–1.4	0.175
2	0.6	0.1–2.6	0.546
3	0	NC	1
VExUS at day 1 (n = 136)			0.955
0	Reference	Reference	Reference
1	1.2	0.4–3.7	0.677
2	0.8	0.1–4.2	0.795
3	NC	NC	1
VExUS at day 2 (n = 111)			0.955
0	Reference	Reference	Reference
1	0.66	0.16–2.8	0.568
2	0.88	0.2–3.2	0.848
3	NC	NC	1

*VExUS* venous ultrasound excess score

## Discussion

In the current study, the results can be summarized as following: (1) the prevalence of systemic venous congestion assessed by the VExUS is low in general ICU patients, (2) VExUS scores did not significantly change during the first days of ICU stay, and (3) systemic venous congestion (VExUS ≥ 2) was not associated with AKI or 28-day mortality in a general ICU cohort.

Our results should be interpreted by taking in account the distinction between moderate and severe congestions that probably do not have the same degree of consequence on kidney function, and by the studied population. The association between systemic venous congestion and kidney failure has been demonstrated in a cardiology setting because the main mechanism of kidney failure may be cardiovascular function. Cardiac surgery patients are more likely to develop venous congestion and AKI because of the high prevalence of associated cardiac disease (systolic or diastolic heart failure, right ventricle failure), and chronic renal failure [21, 22]. Cardiac patients might have more often moderate venous congestion due to chronic disease progression with chronic congestion in the context of neurohormonal alterations (i.e. sympathetic activation, renin–angiotensin–aldosterone system activation, etc.) and hypervolemia [3, 22]. These patients have a high susceptibility to volemia status and they are more prone to develop severe venous congestion, thus AKI [23].

Patients admitted to the ICU for non-cardiac reasons (i.e. septic shock, trauma, stroke) do not necessarily present with a high prevalence of cardiac disease and hypervolemia. These patients have low susceptibility to volemia and are less prone to develop venous congestion. Furthermore, these patients usually develop acute circulatory failure in relation to vasoplegia, hypovolemia, systemic inflammation and bleeding [24]. With recent improvements in hemodynamic care and hemodynamic monitoring, fluid expansion is more often titrated and fluid balance is more carefully taken in account, so fluid overload may now be less frequent than previously observed [25]. The fact that VExUS was not very high and did not significantly change during the first days of ICU admission may indicate that VExUS may not be a good parameter reflecting the volume status in general ICU patients, and that these patients may have low susceptibility to develop severe systemic congestion. The VExUS was previously demonstrated to be not predictive of adequate decongestion by diuretic fluid removal [9].

Another key explanation is relative to the multiple aetiologies of AKI (inflammation, sepsis, anaemia, trauma, iatrogenic) in general ICU patients [20]. AKI has a higher prevalence with more potential causes in a general ICU population than in a specific cardiac surgical cohort [26]. The incidence of AKI in the present cohort is the same as in other multicentric ICU cohorts [20], but it is higher than the incidence reported in cardiac surgery [12]. This aspect might be more true when discussing 28-day mortality [27].

This study illustrates once again the difficulties in validating different parameters developed in cardiac context in other ICU settings, and the continuous struggle to adequately define and assess venous congestion in ICU population. Many studies have suggested that sensitivity and specificity of ultrasound venous congestion markers depend on the severity of congestion, and the studied population. Because many (non-cardiac) factors are associated with AKI in general ICU population, moderate congestion may not be the main determinant that promote AKI. High central venous pressure, particularly in the setting of positive pressure ventilation, might be the reflection of complex cardiopulmonary interactions. Huette et al. [17] demonstrated an impact on portal venous pulsatile flow pattern by using different ventilatory settings. On contrary, when focusing on a population with cardiovascular disease and/or predisposed to heart failure, renal function may be more sensitive to moderate congestion.

### Clinical relevance and perspectives

Systemic venous congestion may not be the main factor for AKI in the general ICU population. As these ultrasound parameters of venous congestion can reflect fluid overload resuscitation, heart failure or pulmonary hypertension, they are specific parameters of venous congestion. Because these parameters have low sensitivity [22], venous congestion diagnosis should not only rely on ultrasound parameters [8]. A combined clinical (oedema, weight, pulmonary crackles, elevated CVP) and ultrasound evaluation should be considered (i.e. cardiac pressure assessment, portal pulsatility index, renal flow patterns), as suggested in cardiology guidelines [22]. Further studies are needed to determine the behaviour of each VExUS component in the context of various ICU therapeutic interventions (invasive or non-invasive ventilation, different levels of positive end-expiratory pressure, different doses and type of vasopressors, etc.) in order to optimise its use and relevance for the diagnosis of congestion in a general ICU setting.

### Limits

This study has several limits. While we analysed a general ICU cohort, our conclusions are restricted to the included patients. General ICU is a broad term that may encompass a vast diversity of conditions; thus, our results should be extrapolated with caution. We also excluded 20% of patients due to incomplete ultrasound data (poor echogenicity, absence of EKG signal, and impossibility to obtain venous ultrasound measurements). Spiegel et al. [10] have observed the same difficulty to assess venous intrarenal flow with ultrasound in a general mixed ICU cohort. Not all the included patients at admission were evaluated later. Excluding patients may be a source of bias, even though we did not detect any specific pattern (i.e. comorbidities, deceased patients in ICU). Our study may be limited by its observational, non-blinded design, and we did not use a direct, quantitative indicator of congestion since it is not possible in current clinical practice. We focused on the first 72 h in ICU, restraining the interpretation to this time window. Other studies have suggested that early hemodynamic parameters cannot be completely extrapolated for later periods in ICU (i.e. acute phase vs de-escalation phase) [28]. We analysed patients with AKI diagnosed at ICU admission (and early during the first hours in ICU). Even though this is close to daily clinical conditions, it might impact the analysis of the relationship between VExUS and AKI, owing to uncertain timing between events. To cope with this issue, we performed a multimodal statistical approach to confirm our findings, and all results being concordant. Finally, the cohort size might not be large enough to detect weaker associations, particularly for severe congestion (i.e. VExUS 3). The low prevalence of severe congestion may be one explanation to the absence of association between systemic venous congestion and AKI. Nevertheless, we used a similar design and sample size as those in previous studies, and the prevalence of systemic congestion was closed to that of previous studies [10, 12, 13].

## Conclusion

In general ICU cohort, the prevalence of moderate to severe systemic venous congestion was low. Early assessment of systemic venous congestion was not associated with AKI or 28-day mortality. Further studies are warranted to define and evaluate the impact of different conditions and therapeutics on the VExUS and its components.

## Supplementary Information

**Additional file 1:** **Table S1.** Baseline characteristics shown comparatively, for patients with admission VExUS ≥ 2 and patients with admission VExUS < 2. **Table S2.** A. Cox regression results with AKI as dependent variable and VExUS at different timepoints as covariate. B. Cox regression results with AKI as dependent variable and VExUS (coded as binary) at different timepoints as covariate. **Table S3.** A. Mixed-effects logistic regression with AKI as dependent variable and VExUS and time as fixed effects, and patient as random effect. B. Mixed-effects logistic regression with AKI as dependent variable and VExUS (coded as binary) and time as fixed effects, and patient as random effect. **Table S4.** A. Cox regression results with 28-day mortality as dependent variable and VExUS at different timepoints as covariate. B. Cox regression results with 28-day mortality as dependent variable and VExUS (coded as binary) at different timepoints as covariate. Figure S1. Study flowchart. **Figure S2.** Alluvial diagram showing the profiles of evolution of VExUS during in ICU. Blue color – patients with admission VExUS ≥ 2. Red color – patients with admission VExUS < 2.

## Data Availability

The datasets used and/or analysed during the current study are available from the corresponding author on reasonable request.

## Abbreviations

AKI: Acute kidney injury
BMI: Body mass index
CW: Continuous wave
CVP: Central venous pressure
HR: Hazard ratio
HV-D: Sus-hepatic vein diastolic flow velocity
HV-S: Sus-hepatic vein systolic flow velocity
ICU: Intensive care unit
IVC: Inferior vena cava
LVEF: Left ventricular ejection fraction
LVOT: Left ventricular outflow tract
MV: Mechanical ventilation
NIV: Non-invasive ventilation
OR: Odds ratio
PI: Portal pulsatility index
PW: Pulsed wave
RVI: Renal venous impedance index
SAPS II: Simplified acute physiological II score
SOFA: Sequential organ failure assessment score
SV: Stroke volume
VExUS: Venous Excess Ultrasound Grading System
Vmax: Maximum velocity
Vmin: Minimum velocity
