# Supplementary material for: Prevalence of systemic venous congestion assessed by Venous Excess Ultrasound Grading System (VExUS) and association with acute kidney injury in a general ICU cohort: a prospective multicentric study
Source: Crit Care. 2023 Jun 8;27:224. doi: 10.1186/s13054-023-04524-4 (PMC10249288; doi:10.1186/s13054-023-04524-4)

**Supplementary Table 1**. Baseline characteristics shown comparatively, for patients with admission VExUS ≥ 2 and patients with admission VExUS < 2.

| **Variables** | **Admission VExUS < 2**  **n = 115** | **Admission VExUS ≥ 2**  **n = 30** | **p-value** |
| --- | --- | --- | --- |
| Age (years), mean (SD) | 64 (15) | 63 (15) | 0.578 |
| Gender, n (%) |  |  |  |
| - Males | 64 (56%) | 22 (73%) | 0.079 |
| Body mass index (kg/m^2^), mean (SD) | 27.4 (6) | 28.2 (7) | 0.791 |
| Comorbidities, n (%) |  |  |  |
| - Chronic obstructive pulmonary disease | 11 (10%) | 3 (10%) | 1 |
| - High blood pressure | 71 (62%) | 15 (50%) | 0.244 |
| - Ischemic heart disease | 36 (31%) | 13 (43%) | 0.215 |
| - Chronic systolic heart failure | 13 (11%) | 8 (27%) | 0.033 |
| - Peripheral artery disease | 10 (9%) | 2 (7%) | 1 |
| - Diabetes on oral antidiabetics | 20 (17%) | 6 (20%) | 0.740 |
| - Diabetes on insulin | 14 (12%) | 1 (3%) | 0.137 |
| - Chronic kidney disease | 11 (10%) | 4 (13%) | 0.376 |
| - Stroke | 8 (7%) | 7 (23%) | 0.009 |
| Medical ICU admission, n (%) | 78 (68%) | 14 (47%) | 0.032 |
| Admission SAPS II, median [IQR] | 46 [34;58] | 49 [40;62] | 0.263 |
| Patients treated with catecholamines, n (%)  Norepinephrine (yes)  Dobutamine (yes) | 49 (42%)  8 (7%) | 14 (47%)  3 (10%) | 0.690  0.406 |
| Abbreviations: ICU – intensive care unit; n – number; SAPS II – simplified acute physiology score II; SD – standard deviation; VExUS – venous excess ultrasound score. | | | |

**Supplementary Table 2A.** Cox regression results with AKI as dependent variable and VExUS at different timepoints as covariate.

| **Variables** | **Hazard ratio** | **95% confidence interval** | **p-value** |
| --- | --- | --- | --- |
| Admission VExUS (n = 145)   - 0 - 1 - 2 - 3 | Reference  0.71  0.59  1.21 | Reference  0.41-1.24  0.26-1.35  0.37-3.95 | 0.429  Reference  0.231  0.216  0.754 |
| VExUS at day 1 (n = 108)^1^   - 0 - 1 - 2 - 3 | Reference  0.72  0.34  0.79 | Reference  0.19-2.72  0.07-1.61  0.1-6.33 | 0.506  Reference  0.631  0.175  0.826 |
| VExUS at day 2 (n = 67)^1^   - 0 - 1 - 2 - 3 | Reference  1.21  0.66  1.56 | Reference  0.61-2.39  0.28-1.52  0.47-5.18 | 0.600  Reference  0.591  0.324  0.465 |
| Abbreviations: VExUS – venous excess ultrasound score. | | | |

^1^ Patients who have developed AKI before the day of ultrasound examination were excluded.

**Supplementary Table 2B.** Cox regression results with AKI as dependent variable and VExUS (coded as binary) at different timepoints as covariate.

| **Variables** | **Hazard ratio** | **95% confidence interval** | **p-value** |
| --- | --- | --- | --- |
| Admission VExUS (n = 145)   - ≥ 2 | 0.94 | 0.27-3.23 | 0.917 |
| VExUS at day 1 (n = 108)^1^   - ≥ 2 | 0.45 | 0.16-1.28 | 0.135 |
| VExUS at day 2 (n = 67)^1^   - ≥ 2 | 0.46 | 0.13-1.64 | 0.231 |
| Abbreviations: VExUS – venous excess ultrasound score. | | | |

^1^ Patients who have developed AKI before the day of ultrasound examination were excluded.

**Supplementary table 3A.** Mixed-effects logistic regression with AKI as dependent variable and VExUS and time as fixed effects, and patient as random effect.

| **Variables** | **Odd ratio** | **95% confidence interval** | **p-value** |
| --- | --- | --- | --- |
| VExUS (all cohort)   - 0 - 1 - 2 - 3 | Reference  0.44  0.19  0.15 | Reference  0.1-5.9  0.1-6.7  0.1-48.5 | Reference  0.535  0.359  0.519 |
| Timepoint   - ICU admission - Day 1 - Day 2 | Reference  0.91  1.26 | Reference  0.11-7.34  0.12-14.4 | Reference  0.925  0.850 |
| Abbreviations: VExUS – venous excess ultrasound score. | | | |

**Supplementary table 3B.** Mixed-effects logistic regression with AKI as dependent variable and VExUS (coded as binary) and time as fixed effects, and patient as random effect.

| **Variables** | **Odd ratio** | **95% confidence interval** | **p-value** |
| --- | --- | --- | --- |
| VExUS (all cohort)   - < 2 - ≥ 2 | Reference  0.3 | Reference  0.1-6.4 | Reference  0.433 |
| Timepoint   - ICU admission - Day 1 - Day 2 | Reference  0.9  1.1 | Reference  0.2-7.2  0.1-12 | Reference  0.919  0.910 |
| Abbreviations: VExUS – venous excess ultrasound score. | | | |

**Supplementary 4A.** Cox regression results with 28-day mortality as dependent variable and VExUS at different timepoints as covariate.

| **Variables** | **Hazard ratio** | **95% confidence interval** | **p-value** |
| --- | --- | --- | --- |
| Admission VExUS (n = 145)   - 0 - 1 - 2 - 3 | Reference  0.53  0.82  0 | Reference  0.2-1.5  0.23-3  NC | 0.699  Reference  0.232  0.768  1 |
| VExUS at day 1 (n = 136)^1^   - 0 - 1 - 2 - 3 | Reference  1.2  0.96  0 | Reference  0.45-3.3  0.2-4.5  NC | 0.982  Reference  0.704  0.963  1 |
| VExUS at day 2 (n = 111)^1^   - 0 - 1 - 2 - 3 | Reference  0.88  1.16  0 | Reference  0.24-3.18  0.36-3.69  NC | 0.987  Reference  0.84  0.805  1 |
| Abbreviations: VExUS – venous excess ultrasound score. | | | |

^1^ Patients who have been deceased before the day of ultrasound examination were excluded.

**Supplementary Table 4B.** Cox regression results with 28-day mortality as dependent variable and VExUS (coded as binary) at different timepoints as covariate.

| **Variables** | **Hazard ratio** | **95% confidence interval** | **p-value** |
| --- | --- | --- | --- |
| Admission VExUS (n = 145)   - ≥ 2 | 0.82 | 0.41-1.6 | 0.552 |
| VExUS at day 1 (n = 136)^1^   - ≥ 2 | 0.98 | 0.48-2 | 0.983 |
| VExUS at day 2 (n = 111)^1^   - ≥ 2 | 0.75 | 0.38 -1.51 | 0.42 |
| Abbreviations: VExUS – venous excess ultrasound score. | | | |

^1^ Patients who have been deceased before the day of ultrasound examination were excluded.

.

**Supplemental Figure 1. Study flowchart**


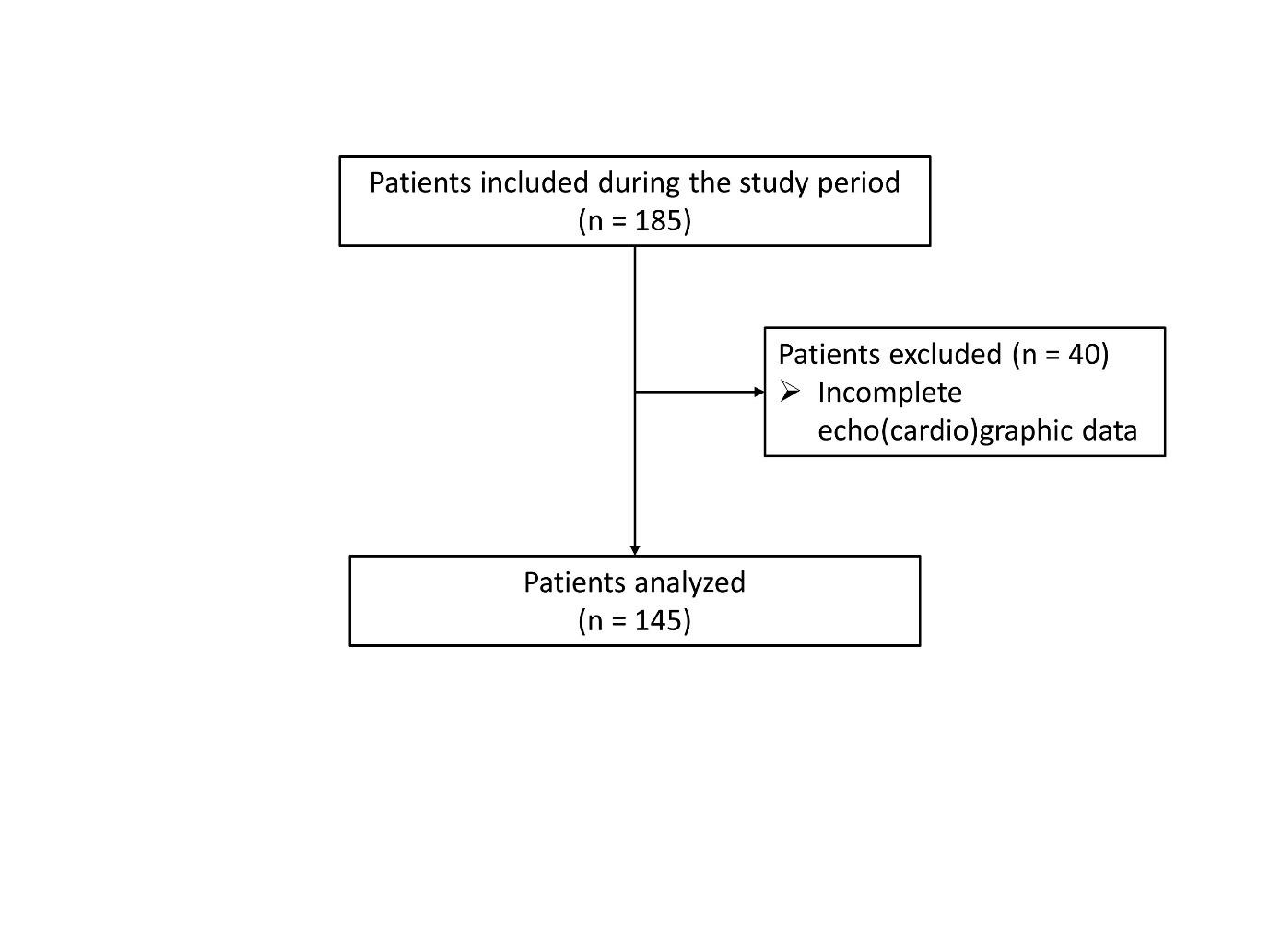


**Supplemental Figure 2.** Alluvial diagram showing the profiles of evolution of VExUS during in ICU. Blue color – patients with admission VExUS ≥ 2. Red color – patients with admission VExUS < 2.


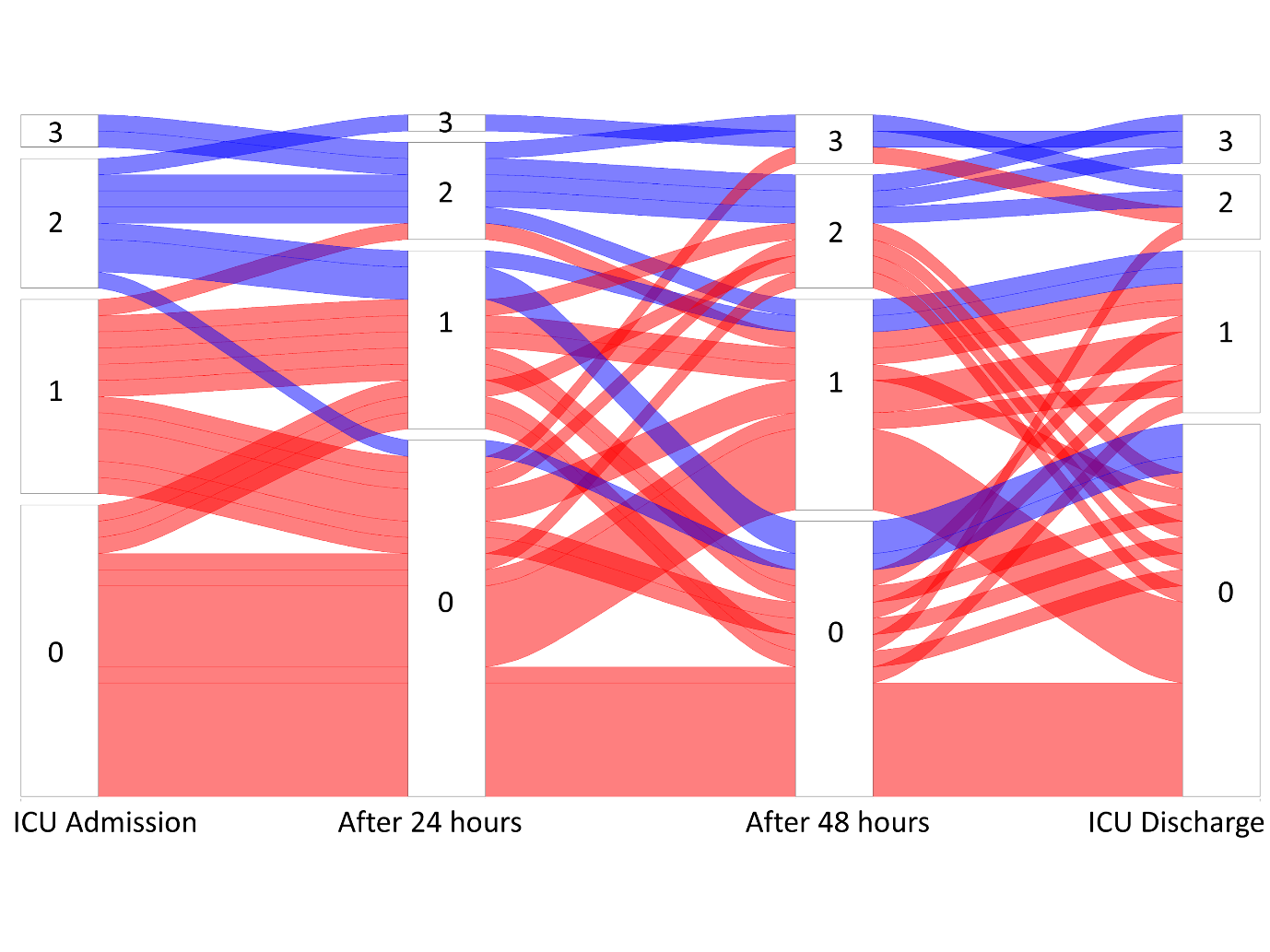

Supplement: Supplementary file 1 — Additional file 1: Table S1. Baseline characteristics shown comparatively, for patients with admission VExUS ≥ 2 and patients with admission VExUS < 2. Table S2. A. Cox regression results with AKI as dependent variable and VExUS at different timepoints as covariate. B. Cox regression results with AKI as dependent variable and VExUS (coded as binary) at different timepoints as covariate. Table S3. A. Mixed-effects logistic regression with AKI as dependent variable and VExUS and time as fixed effects, and patient as random effect. B. Mixed-effects logistic regression with AKI as dependent variable and VExUS (coded as binary) and time as fixed effects, and patient as random effect. Table S4. A. Cox regression results with 28-day mortality as dependent variable and VExUS at different timepoints as covariate. B. Cox regression results with 28-day mortality as dependent variable and VExUS (coded as binary) at different timepoints as covariate. Figure S1. Study flowchart. Figure S2. Alluvial diagram showing the profiles of evolution of VExUS during in ICU. Blue color – patients with admission VExUS ≥ 2. Red color – patients with admission VExUS < 2. [file 13054_2023_4524_MOESM1_ESM.docx]
